# Risk-stratified surveillance and cost effectiveness of follow-up after radical cystectomy in patients with muscle-invasive bladder cancer

**DOI:** 10.18632/oncotarget.19043

**Published:** 2017-07-06

**Authors:** Ayumu Kusaka, Shingo Hatakeyama, Shogo Hosogoe, Itsuto Hamano, Hiromichi Iwamura, Naoki Fujita, Ken Fukushi, Takuma Narita, Kazuhisa Hagiwara, Hayato Yamamoto, Yuki Tobisawa, Tohru Yoneyama, Takahiro Yoneyama, Yasuhiro Hashimoto, Takuya Koie, Hiroyuki Ito, Kazuaki Yoshikawa, Toshiaki Kawaguchi, Chikara Ohyama

**Affiliations:** ^1^ Department of Urology, Hirosaki University Graduate School of Medicine, Hirosaki, Japan; ^2^ Department of Advanced Transplant and Regenerative Medicine, Hirosaki University Graduate School of Medicine, Hirosaki, Japan; ^3^ Department of Urology, Aomori Rosai Hospital, Hachinohe, Japan; ^4^ Department of Urology, Mutsu General Hospital, Mutsu, Japan; ^5^ Department of Urology, Aomori Prefectural Central Hospital, Aomori, Japan

**Keywords:** medical cost, radical cystectomy, recurrence, screening, surveillance

## Abstract

**Background:**

The recurrence risk stratification and the cost effectiveness of oncological surveillance after radical cystectomy are not clear. We aimed to develop a risk stratification and a surveillance protocol with improved cost effectiveness after radical cystectomy.

**Results:**

Of 581 enrolled patients, 175 experienced disease recurrences. The pathology-based protocol presented significant differences in recurrence-free survival between normal- and high-risk patients, but the medical expense was high, especially in normal-risk (≤pT2pN0) patients. Cox regression analysis identified six factors associated with recurrence-free survival. Risk score-based 5-year follow-up was significantly more cost effective than the pathology-based protocol.

**Materials and Methods:**

We retrospectively evaluated 581 patients with radical cystectomy for muscle-invasive bladder cancer at 4 hospitals. Patients with routine oncological follow-up were stratified into normal- and high-risk groups by a pathology-based protocol utilizing pT, pN, lymphovascular invasion, and histology. Cost effectiveness of the pathology-based protocol was evaluated and a risk-score-based protocol was developed to optimize cost effectiveness. Risk-scores were calculated by summing risk factors independently associated with recurrence-free survival. Patients were stratified by low-, intermediate-, and high-risk score. Estimated cost per one recurrence detection by the pathology and by risk-scores were compared.

**Conclusions:**

Risk-score-stratified surveillance protocol has potential to reduce over-evaluation after radical cystectomy without adverse effects on medical cost.

## INTRODUCTION

Radical cystectomy (RC) with extended pelvic lymph node dissection is the standard treatment for non-metastatic muscle-invasive bladder cancer (MIBC) [1, 2]. Long-term outcome and predictors of disease relapse after RC are well documented [1–4]. Despite advances in treatment, an estimated 38%–49% of patients experience recurrence within 10 years even at high-volume institutes [1, 2, 4]. The prognosis is dismal following recurrence. Various surveillance regimens have been proposed [4–7], but the most effective follow-up frequency and methods of evaluation are unclear. In addition, evidence is lacking on the cost effectiveness of routine oncological follow-up to detect recurrence after RC [4, 8] and needs investigation. The main purposes of the present study are to develop the risk stratification and the optimal surveillance protocol for tumor recurrence that improves cost effectiveness after RC. First, we estimated the per-capita cost of detecting post-RC recurrence of MIBC using an existing pathology-based protocol (Table 1, upper rows). We then developed a novel risk-score-based protocol using Cox proportional hazard regression and compared the cost effectiveness of the pathology-based and risk-score-based protocols.

**Table 1:** Pathology-based and risk-score-based protocol for oncological follow-up

Pathology-based protocol	Months after RC													
Normal-risk	3	6	9	12	15	18	21	24	30	36	42	48	54	60
Ultrasonography	●	●	●	●	○	●		●	●	●	●	●	●	●
Urine cytology	●	●	●	●	○	●		●		●		●		●
Blood and serum test	●	●		●		●		●		●		●		●
CT scan		●		●		●		●		●		●		●
High-risk	3	6	9	12	15	18	21	24	30	36	42	48	54	60
Ultrasonography	●	●	●	●	●	●	●	●		●		●		●
Urine cytology	●	●	●	●	●	●	●	●		●		●		●
Blood and serum test	●	●	●	●	●	●	●	●		●		●		●
CT scan	●	●	●	●	●	●	●	●	●	●	●	●	●	●

Risk-score-based protocol	Months after RC													
Low-risk	3	6	9	12	15	18	21	24	30	36	42	48	54	60
Ultrasonography		●		●				●		●		●		●
Urine cytology		●		●				●		●		●		●
Blood and serum test		●		●				●		●		●		●
CT scan				●						●				
Intermediate- and high-risk	3	6	9	12	15	18	21	24	30	36	42	48	54	60
Ultrasonography	●	●	●	●		●		●		●		●		●
Urine cytology	●	●	●	●		●		●		●		●		●
Blood and serum test	●	●	●	●		●		●		●		●		●
CT scan	●	●	●	●	●	●	●	●		●		●*		●

*,only for the high-risk group, CT scan included chest, abdomen and pelvis. ○, optional examination when indicated.

## RESULTS

**Figure 1:**
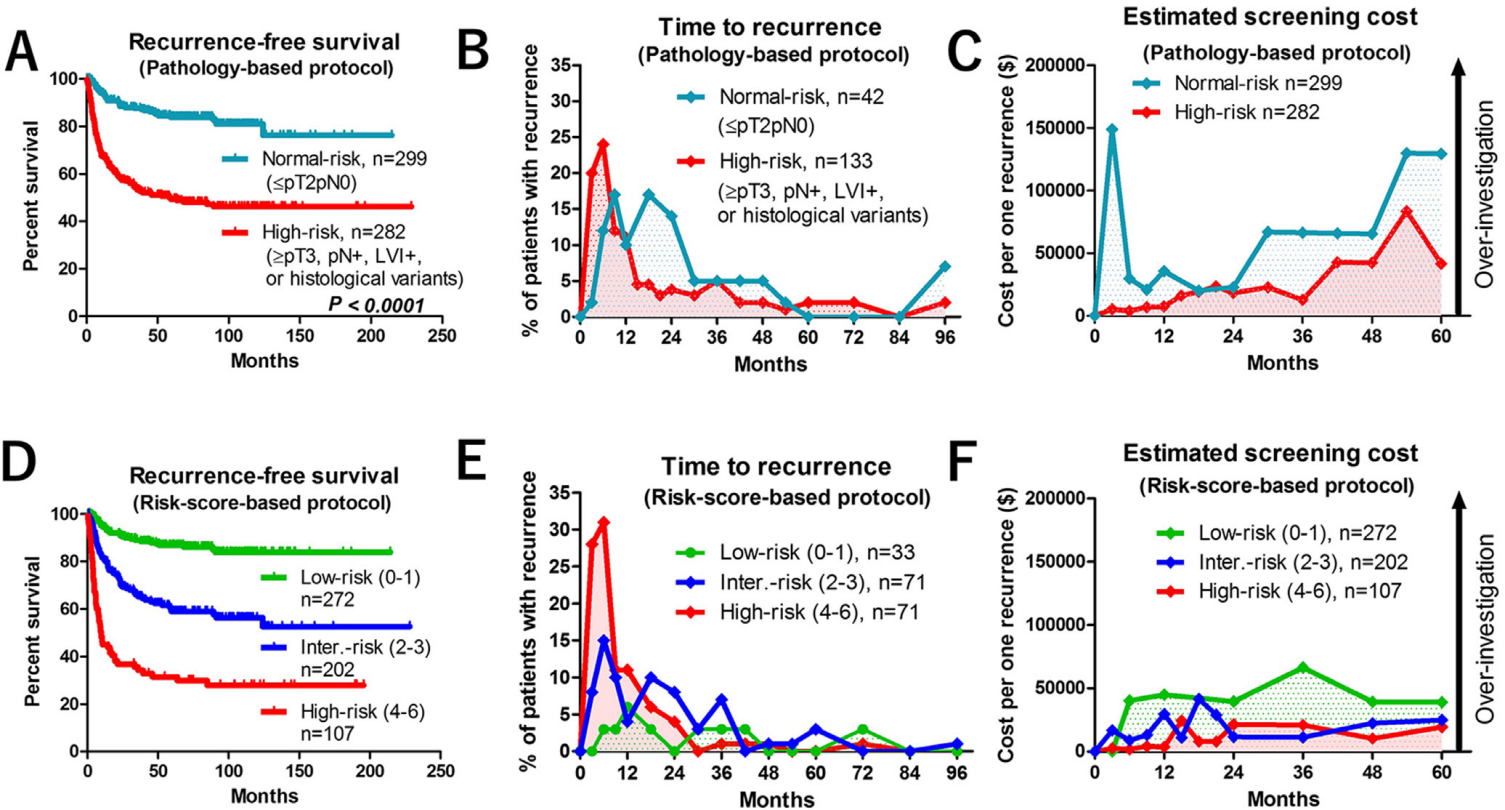
Oncological and economic outcomes of pathology-based and risk score-based surveillance protocols (**A**) Recurrence-free survival in normal-risk (≤ pT2pN0) and high-risk (≥ pT3, pN+, lymphovascular invasion [LVI+], or histological variants) patients in the pathology-based protocol. (**B**) Time-course analysis of recurrence pattern of normal-risk and high-risk patients in the pathology-based protocol. (**C**) Estimated cost per one recurrence detection in normal-risk and high-risk patients in the pathology-based protocol. (**D**) Recurrence-free survival of low-risk score (score 0–1), intermediate-risk score (2–3), and high-risk score (4–6) patients. (**E**) Time-course analyses of recurrence in low-risk, intermediate-risk, and high-risk score patients. (**F**) Estimated cost per one recurrence detection in low-risk, intermediate-risk, and high-risk score patients.

**Figure 2:**
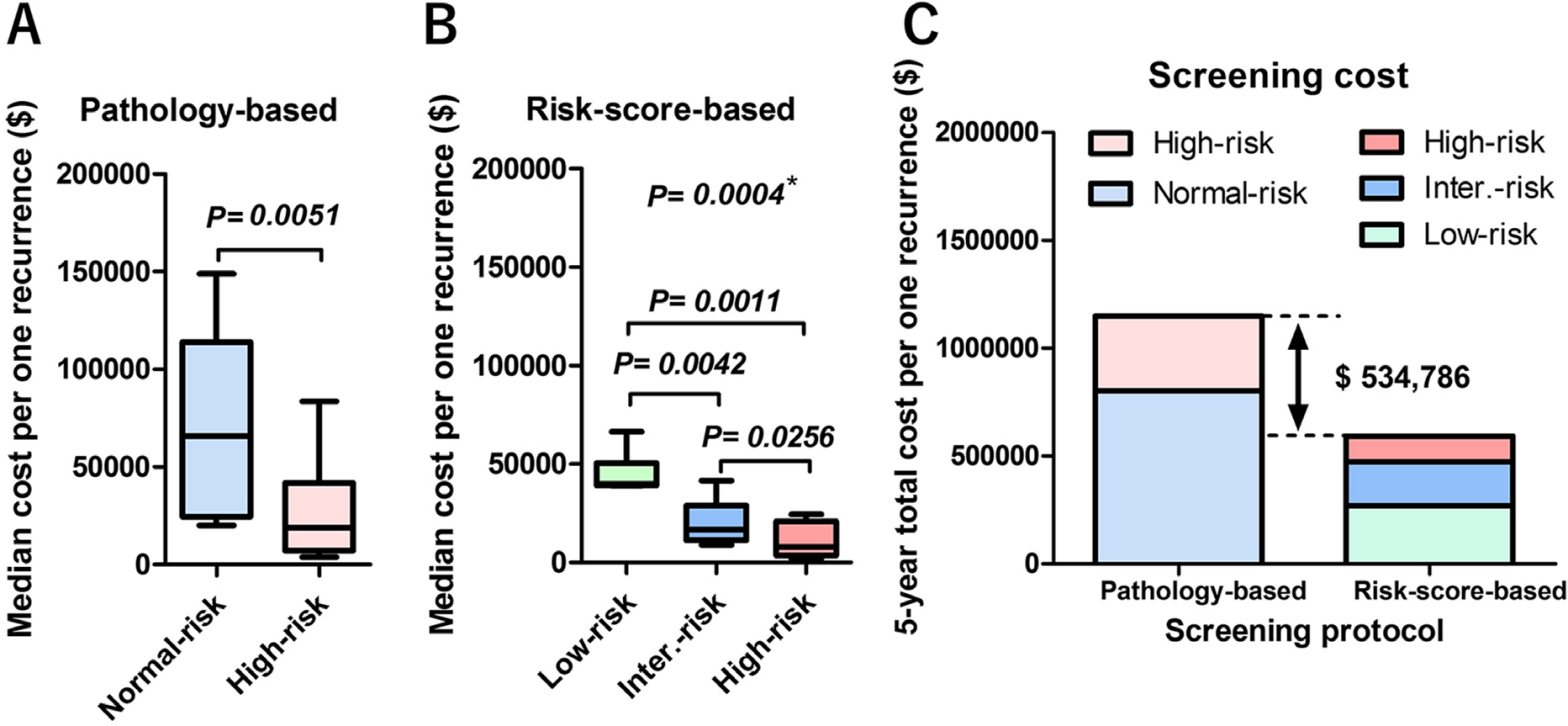
Per-capita cost to detect 5-year recurrence (**A**) In the pathology-based protocol, median screening cost to detect one recurrence in 5 years was significantly higher in the normal-risk ($65,736) group than that of high-risk ($18,775) group (*P* = 0.0051). (**B**) In the risk score-based protocol, median screening cost to detect one recurrence in 5 years was significantly different in the three groups (*, Kruskal-Wallis test). There were significant differences between the low-risk ($39,960) and intermediate-risk ($16,766) groups (*P* = 0.0042), the low-risk and high-risk ($10,209) groups (*P* = 0.0011), and the intermediate-risk and high-risk groups (*P* = 0.0256). (**C**) The estimated 5-year screening cost in the pathology-based protocol was $1,148,687, whereas it was $613,901 in the risk score-based protocol. The estimated difference was $534,786 over 5 years.

Of the 581 patients with RC, 299 (52%) were included in the pathology-based normal-risk group and 282 (48%) were included in the high-risk group. The clinicopathological characteristics are shown in Table 2. Patient sex ratio, preoperative eGFR, ≥ cT3, surgical margin positive (SM+), cN+, neoadjuvant chemotherapy (NAC), type of urinary diversion, and tumor recurrence in the normal- and high-risk groups were significantly different. Because of a higher rate of relapse, median follow-up was significantly shorter in the high-risk (33 months) than in the normal-risk (60 months) group. Recurrence-free survival in the 2 groups was also significantly different (Figure 1A). The number of patients with symptomatic tumor recurrence was 100/175 (57%). There was no significant difference in the number of patients with symptomatic recurrence between the normal-risk 28/43 (65%) and high-risk 72/132 (55%) groups (*P* = 0.291). The number of patients with tumor recurrence within 24 months in the high-risk group (*n* = 109/133, 82%) and the normal-risk group (n = 30/42, 71%) were not significantly different (*P* = 0.189, Figure 1B). The estimated cost per one recurrence detection was extremely high at 3, 54, and 60 months (> $100,000) in the normal-risk group (Figure 1C). The 12-month cost effectiveness was more acceptable in the high-risk group (< $10,000), but gradually increased thereafter (Figure 1C). The median estimated cost per one recurrence detection was significantly higher in the normal-risk ($65,736) group than in the high-risk ($18,775) group (*P* = 0.0051) (Figure 2A)

**Table 2:** Background of patients in pathology-based protocol

	All	Normal-risk	High-risk	*P* value
*n*	581	299	282	
Age, years (IQR)	69 (62–75)	69 (62–74)	69 (62–75)	*0.694*
Sex (Male), *n =*	458 (79%)	247 (83%)	221 (75%)	*0.022*
ECOG PS > 0, *n =*	15 (2.6%)	5 (1.7%)	6 (2.1%)	*0.767*
Hypertension (HTN), *n =*	179 (31%)	92 (31%)	85 (30%)	*0.870*
Diabetes Mellitus (DM), *n =*	78 (13%)	47 (16%)	30 (11%)	*0.425*
Cardiovascular disease (CVD), *n =*	68 (12%)	25 (8.4%)	29 (10%)	*0.071*
Preoperative CKD, *n =*	215 (37%)	122 (41%)	131 (46%)	*0.170*
Preoperative eGFR (mL/min/1.73m^2^)	66 ± 20	68 ± 20	63 ± 19	*0.010*
≥cT3, *n =*	274 (47%)	101 (34%)	173 (61%)	*< 0.001*
Surgical margin positive (SM+), *n =*	26 (4.5%)	0 (0%)	26 (9.2%)	*< 0.001*
cN positive, *n =*	44 (7.6%)	12 (4.0%)	32 (11%)	*0.001*
Neoadjuvant chemotherapy (NAC), *n =*	315 (60%)	197 (66%)	151 (54%)	*0.002*
Urinary diversion (Neobladder), *n =*	315 (54%)	195 (75%)	123 (44%)	*< 0.001*
Postoperative complication (Grade 3 or higher)	15 (2.6%)	8 (1.4%)	7 (1.2%)	*1.000*
Non-urothelial carcinoma components included, *n =*	37 (6.4%)	0 (0%)	37 (13%)	
≥ pT3, *n =*	183 (31%)	0 (0%)	181 (64%)	
pN positive, *n =*	70 (12%)	0 (0%)	79 (28%)	
Lympho-vascular invasion (LVI), *n =*	195 (34%)	0 (0%)	194 (69%)	
Tumor recurrence, *n =*	175 (30%)	43 (14%)	132 (47%)	*< 0.001*
Median follow-up, months (IQR)	45 (13–90)	60 (23–95)	33 (9.7–75)	*< 0.001*

CKD: chronic kidney disease, SM: surgical margin.

**Figure 3:**
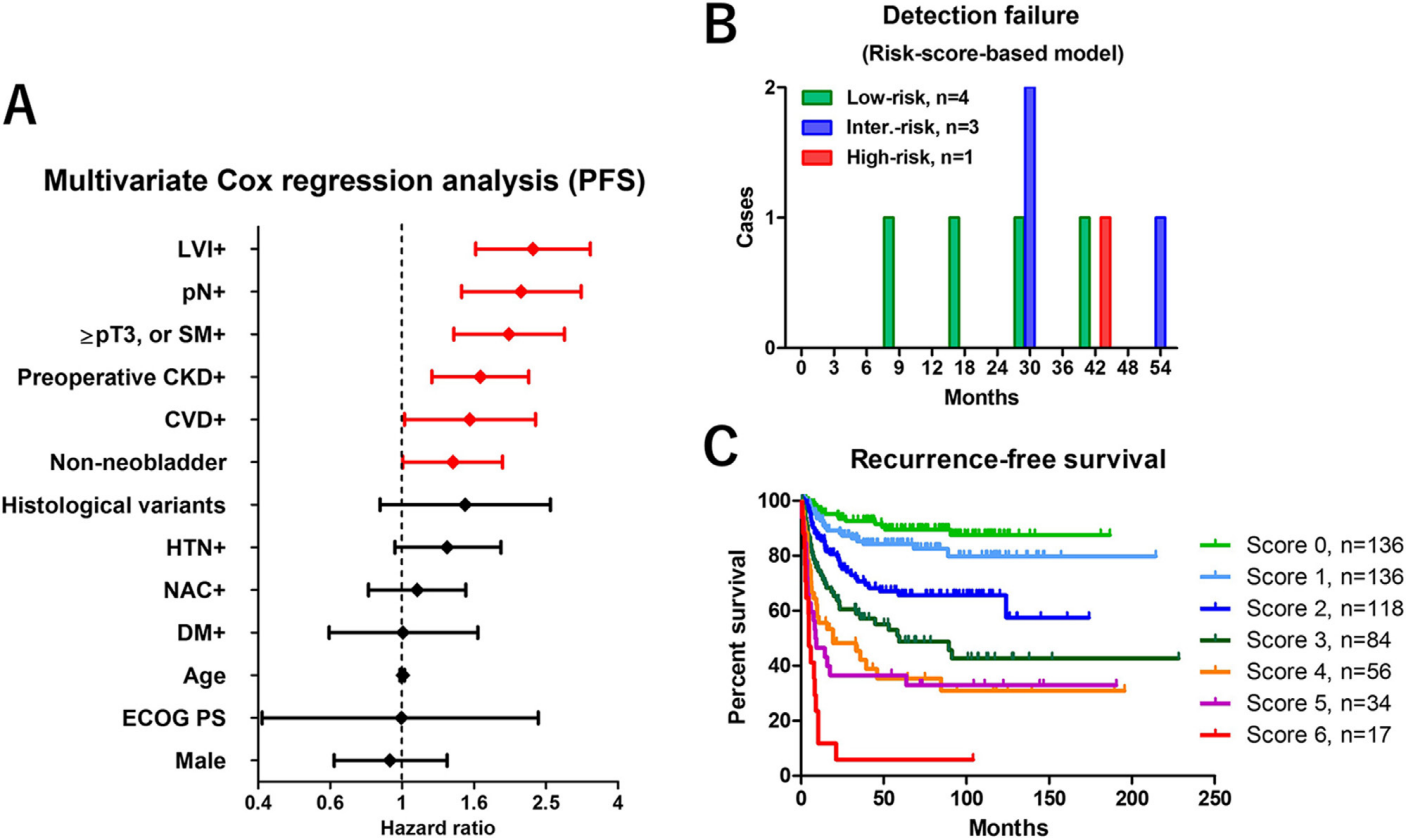
Risk factors selection and the impact of risk-scores on prognosis (**A**) Hazard ratio and 95% CI in multivariate Cox regression analysis. (**B**) The number of patients potentially undetected by the risk score based protocol were 4 (12%) in the low-, 3 (4.2%) in the intermediate- and 1 (1.4%) in the high-risk group. (**C**) Recurrence-free survival was shown in each risk score. Patients were stratified into three groups by low-risk score (0–1), intermediate-risk score (2–3), and high-risk score (4–6), indicating the probability of relapse. LVI: lymphovascular invasion, SM: surgical margin, CKD: chronic kidney disease, CVD: cardiovascular disease, HTN: hypertension, NAC: neoadjuvant chemotherapy, DM: diabetes mellitus

Multivariate Cox regression analysis found 6 factors that were independently associated with recurrence-free survival, including LVI+, pN+, ≥pT3 or SM+, urinary diversion (non-neobladder), preoperative chronic kidney disease (CKD), and cardiovascular disease (CVD) (Table 3, Figure 3A). The risk-scores were calculated by adding each independent risk factor for recurrence-free survival (risk score ranged 0–6). Patients were divided by their score into low-risk (0–1), intermediate-risk (2–3), and high-risk (4–6) groups (Table 4). Recurrence-free survival was significantly shorter in patients with high-risk than with low-risk (*P* < 0.001) or intermediate-risk (*P* < 0.001) scores (Figure 1D). The number of patients who experienced tumor recurrence within 24 months was significantly higher in high-risk-score (n = 65/71, 92%) than in the low-risk score (n = 5/33, 15%, *P* < 0.001) or intermediate-risk score (n = 40/71, 56%, *P* < 0.001) groups (Figure 1E). The number of patients with symptomatic tumor recurrence in the low-risk-score, intermediate-risk score, and high-risk score were 18/33 (55%), 41/71 (58%), and 41/71 (58%), respectively. Based on this risk-stratification, we developed a risk score-based protocol for oncological follow-up (Table 1, lower rows), and evaluated per-capita cost of recurrence detection (Figure 1F). The median estimated cost per one recurrence detection was significantly different between the low-risk ($39,960) and intermediate-risk ($16,766) groups (*P* = 0.0042), the low-risk and high-risk ($10,209) groups (*P* = 0.0011), and the intermediate-risk and high-risk, groups (*P* = 0.0256) (Figure 2B). The risk-score-based protocol led to a dramatic cost reduction compared to the pathology-based protocol. The total estimated 5-year screening cost was 1.9-fold higher with the pathology-based protocol ($1,148,687) than with the risk score-based protocol ($613,901). The estimated cost difference was $534,786 for 5 years (Figure 2C). The number of patients that potentially fails in detection using the risk-score-based protocol was 4 (12%) in the low, 3 (4.2%) in the intermediate, and 1 (1.4%) in the high-risk-score group (Figure 3B).

**Table 3:** Multivariate analysis for progression-free survival

	Risk	*P* value	HR	95% CI
Age	Continuous	*0.550*	*1.01*	0.99–1.02
Sex	Male	*0.703*	*0.93*	0.65–1.34
ECOG PS	> 0	*0.995*	*1.00*	0.41–2.40
Hypertension (HTN)	Positive	*0.090*	*1.34*	0.96–1.89
Diabetes Mellitus (DM)	Positive	*0.969*	*1.01*	0.63–1.63
Cardiovascular disease (CVD)	Positive	*0.042*	*1.55*	1.02–2.36
Preoperative CKD	Positive	*0.001*	*1.66*	1.22–2.26
Neoadjuvant chemotherapy (NAC)	Underwent	*0.526*	*1.11*	0.81–1.51
Histology	Variants included	*0.142*	*1.50*	0.87–2.59
Urinary diversion	Non–neobladder	*0.049*	*1.39*	1.00–1.92
Pathological T stage	≥ pT3 or SM+	*< 0.001*	*1.99*	1.40–2.84
Pathological N stage	pN positive	*< 0.001*	*2.15*	1.47–3.16
Lymphovascular invasion	Positive	*< 0.001*	*2.32*	1.61–3.35

CKD: chronic kidney disease, SM: surgical margin

**Table 4:** Risk-score-based classification

Variable	Status	Risk score
Cardiovascular disease	Positive	1
Preoperative CKD	Positive	1
Urinary diversion	Non-neobladder	1
Pathological T stage	≥ pT3 or SM+	1
Pathological N stage	pN positive	1
Lymphovascular invasion	Positive	1

Risk-score-based classification		Sum of risk-score
Low-risk		0–1
Intermediate-risk		2–3
High-risk		4–6

CKD: chronic kidney disease, SM: surgical margin.

## DISCUSSION

Currently, there is no strong evidence or consensus on how to appropriately follow-up RC patients after surgical intervention [9], and a few studies have been conducted to investigate the cost effectiveness of surveillance regimens after RC [8]. The effectiveness of a stage-based approach to tumor surveillance after RC has been shown [10, 11]. Yafi et al. reported the effectiveness of a stage-based protocol (≤ pT2pN0, pT3-4pN0, pTxpN+) that captured most recurrences while limiting over investigation using pooled data from 2287 patients in 8 Canadian academic centers [4]. Although pathological stage is known to be a strong predictor of relapse, variations of recurrence pattern in those patients prevented effective screening with a universal surveillance protocol for all patients. Our results showed that a pathology-based screening protocol has potential to increase unnecessary testing in normal-risk (≤ pT2pN0) patients. Not only pathological outcome but other clinical risk factors have significant impact on tumor recurrence after RC. To address this, we first estimated the cost per one recurrence detection using a pathology-based protocol, which was found not optimal as a way to stratify patient recurrence risk for cost effective surveillance. We developed risk-score-based surveillance protocol and stratified the study patients into low-risk (0–1), intermediate-risk (2–3) and high-risk (4–6) score groups. The recurrence-free survival in each risk-score is shown in Figure 3C. Not only pathological outcomes, but also preoperative variables including CVD and CKD had a significant impact on recurrence after RC. Recent studies suggested the impact of CKD on prognosis in MIBC [12] and upper urinary tract urothelial cancer [13]. Patients with renal impairment are at risk of CVD [14], but the reason for the strong association between CKD and poor recurrence-free survival is not clear. Previous studies suggested kidney and urinary tract cancers are high risk for renal dysfunction due to the tumor location (obstruction, and/or reduction of nephron mass) [13, 15–17]. The prevalence of CKD in genitourinary cancers were reported 8.7–21.4% [15, 18]. In the present study, the prevalence of CKD in MIBC patients was 37%, which is a strong bias in our cohort. We investigated the relationship between CKD, CVD and prognosis. Our results suggested patients with CKD had significantly higher risk for recurrence and cancer death than the patients without CKD. Similarly, patients with CVD had significantly higher risk for recurrence and cancer death than the patients without CVD (Figure 5A, 5B). In addition, the patients with CKD had significantly higher risk-score (2.5 ± 1.3 points) than those without CKD (1.2 ± 1.3 points, *P* < 0.001) (Figure 5C, left). Similarly, the patients with CVD had significantly higher risk-score (3.1 ± 1.3 points) than those without CVD (1.6 ± 1.4 points, *P* < 0.001) (Figure 5C, right). However, when simply compared cause of death, no difference was observed in the cause of death between patients with and without CKD (Figure 5D). In addition, there was no significant difference between the number of CKD patients with CVD (n=35/68, 52%) and without CVD (*n* = 218/513, 42%) (Figure 5E). Therefore, as previous study suggested [18], bladder cancer is great potential to have a renal dysfunction during the disease progression. Although there is no clear explanation for that, the effects of metabolic syndrome [19] and chronic inflammation may explain the association of CKD and oncological outcome [20, 21]. Long-term inflammation and oxidative stress caused by CKD and linked to organ degradation may increase carcinogenicity. The other potential factor that influencing on poor prognosis was frailty. Patients with CKD and CVD are more likely to be frail [22] because these are likely to exist in combination with comorbid conditions, disability, and polypharmacy [23, 24]. In addition, frailty is also one of the important parameters of cancers [25, 26]. Recent study reported the association between frailty and inflammatory markers in elderly cancer patients [27]. Our previous study suggested renal function has potential to predict postoperative frailty [28]. Although we could not address the direct reason why CKD and CVD were independently influence on recurrence after RC, these results suggest a potential relationship between cancer progression, CKD, CVD and frailty. However, evidence to confirm this hypothesis is lacking. Future studies to assess mechanisms underlying carcinogenesis and risk factors are warranted.

**Figure 4:**
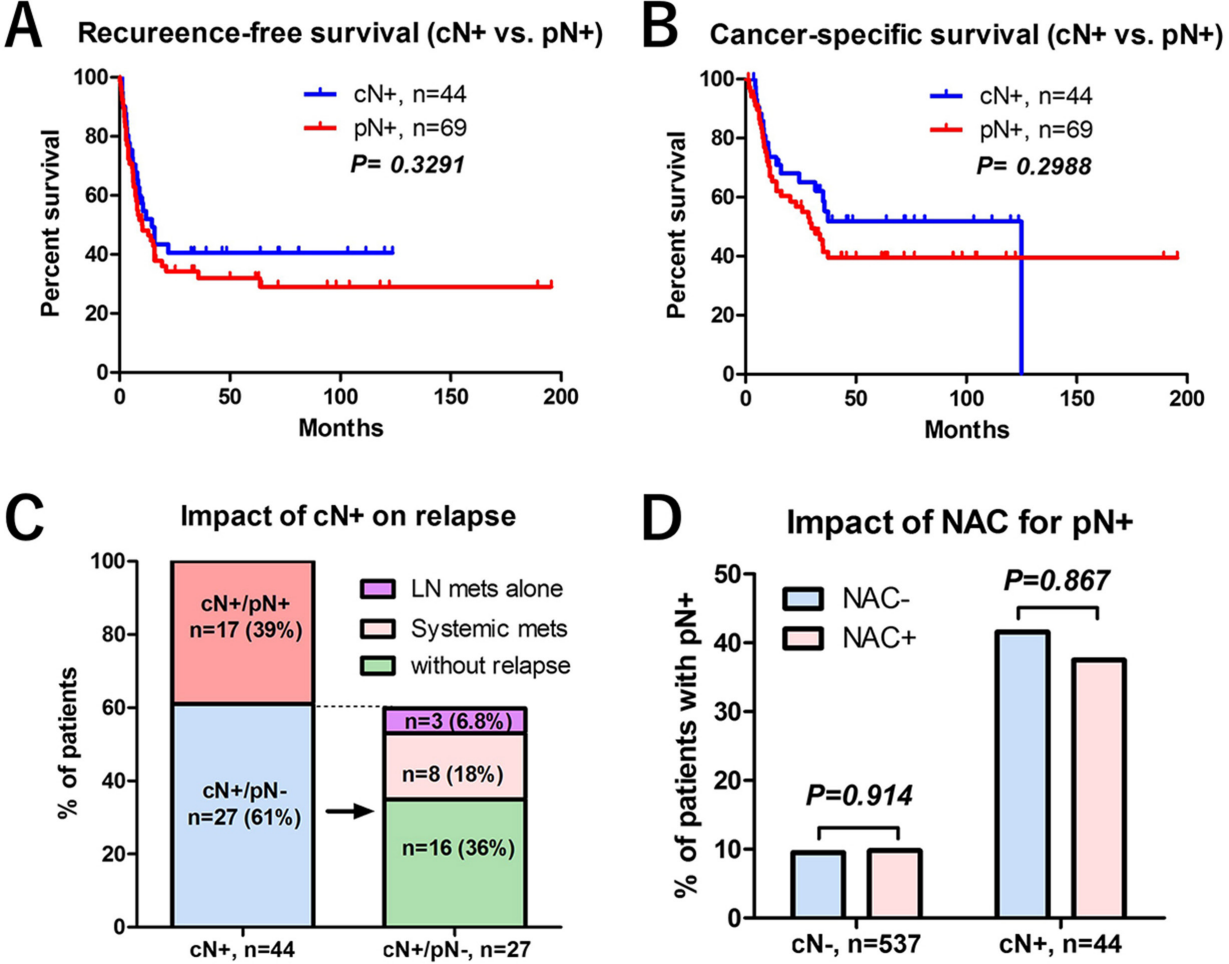
The impact of lymph node positive (cN+ or pN+) on prognosis (**A**) Recurrence-free survival between the patients with cN+ and pN+ was not significantly different. (**B**) Cancer-specific survival between the patients with cN+ and pN+ was not significantly different. (**C**) The impact of cN+ on tumor recurrence was shown. Of 44 patients with cN+, only 17 (39%) patients had cN+ and pN+. Of 27 (61%) patients with cN+/pN-, 11 patients (25%) experienced recurrence. Three patients with cN+/pN- (6.8%) had lymph nodes recurrence alone while remain 8 (18%) had systemic recurrence. (**D**) There were no significant difference in the frequencies of pN+ between cN- patients treated with NAC (31/316, 9.8%) and without NAC (21/221, 9.5%) (*P* = 0.914), and pN+ between cN+ treated with NAC (12/32, 38%) and without NAC (5/12, 42%) (*P* = 0.867).

**Figure 5:**
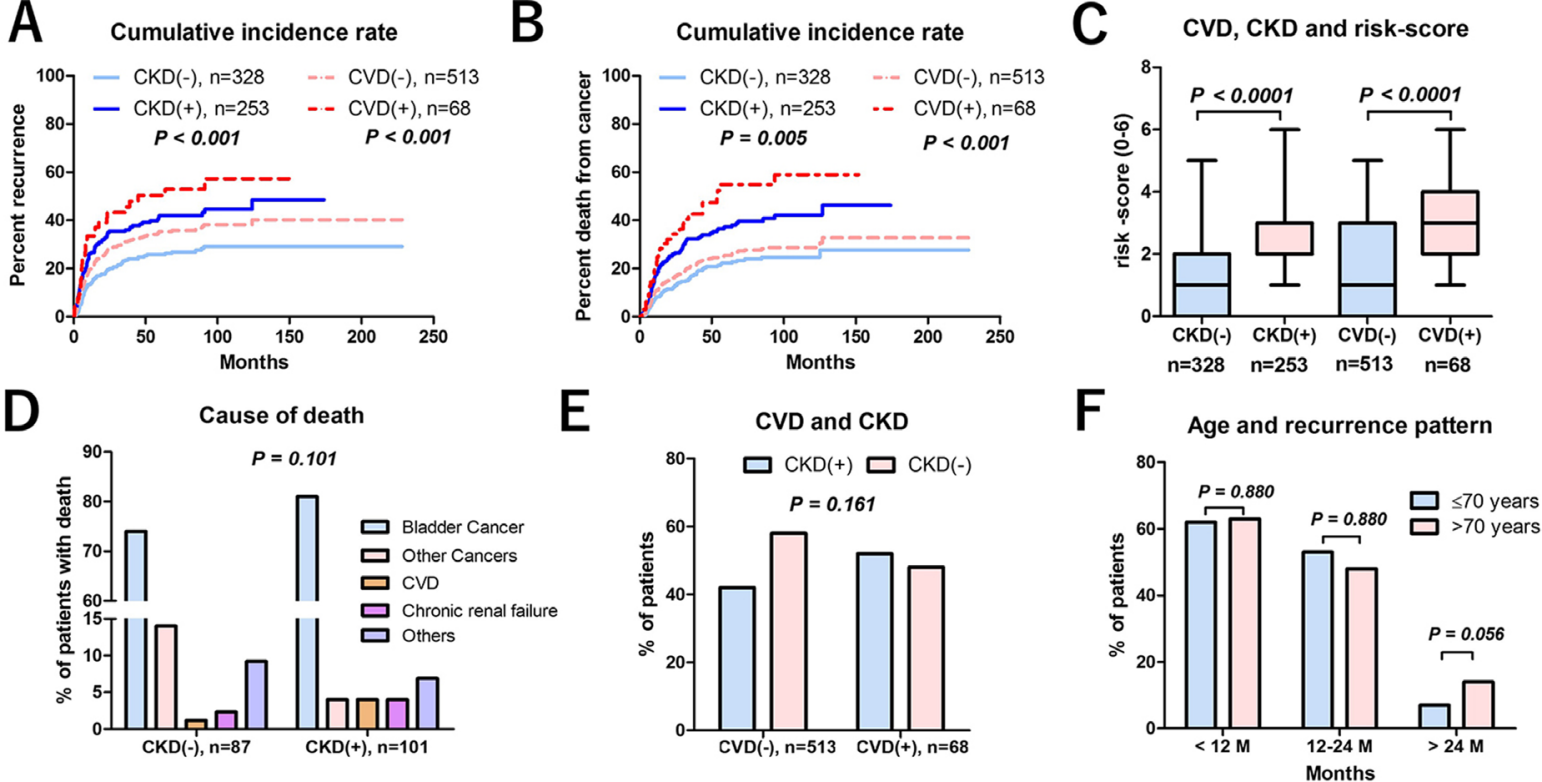
The impact of chronic kidney disease (CKD), cardiovascular disease (CVD), or age on prognosis (**A**) Recurrence-free survival of the patients with CKD was significantly shorter than the patients without CKD. Recurrence-free survival in the patients with CVD was significantly shorter than the patients without CVD. (**B**) Cancer-specific survival of the patients with CKD was significantly shorter than the patients without CKD. Cancer-specific survival in the patients with CVD was significantly shorter than the patients without CVD. (**C**) The patients with CKD had significantly higher risk-score (2.5 ± 1.3 points) than those without CKD (1.2 ± 1.3 points, *P* < 0.001) (Figure 5C, left). Similarly, the patients with CVD had significantly higher risk-score (3.1 ± 1.3 points) than those without CVD (1.6 ± 1.4 points, *P* < 0.001) (Figure 5C, right). (**D**) No difference was observed in the cause of death between patients with and without CKD. (**E**) There was no significant difference between the number of CKD patients with CVD (*n* = 35/68, 52%) and without CVD (*n* = 218/513, 42%). (**F**) The relationship between age and recurrence pattern showed no significantly difference between the patients with ≤ 70 and with > 70 years old.

Age is also a well-known risk factor for recurrence. To investigate the relationship between age and recurrence pattern, we divided patients into two groups between younger (≤ 70) and older (> 70). Our results suggested that there was no significant difference in the recurrence pattern between the younger (≤ 70) and older (> 70) patients. However, late recurrence (> 24 months) showed marginal difference between ≤ 70 years (15/202, 7%) and > 70 years (19/138, 14%) (*P* = 0.056) (Figure 5F).

Inclusion of cN+ for risk-stratification needs to be debated. Because standard lymph node dissection is recommended for all patients at RC, we selected pN+ for one of the risk factors. Although cN+ is one of the risk factors for poor prognosis (Figure 4A, 4B, and Table 3), the clinical implication of cN+ remains unclear because not all cN+ predicts tumor relapse. In the present study, only 17/44 (39%) patients with cN+ had pN+ by standard lymph node dissection. Of 27/44 (61%) patients with cN+/pN-, 11 patients (25%) experienced recurrence. Three patients with cN+/pN- (6.8%) had lymph nodes recurrence alone while remain 8 (18%) had systemic recurrence (Figure 4C). On the other hand, 52/537 (9.7%) patients with cN- had pN+ by the standard lymph node dissection. In addition, the influence of NAC for pN status need to be addressed. We evaluated the difference of pN status between cN- patients treated with NAC and without NAC, and pN status between cN+ treated with NAC and without NAC. The result showed there were no significant difference of pN+ between cN- patients treated with NAC (31/316, 9.8%) and without NAC (21/221, 9.5%) (P=0.914), and pN+ between cN+ treated with NAC (12/32, 38%) and without NAC (5/12, 42%) (*P* = 0.867) (Figure 4D). These results suggested the inaccuracy of cN+ to predict pathological lymph node involvement, although we could not exclude false negative of standard lymph node dissection. Based on these limitations, we included pN+ for the risk-stratification in the present study. However, further studies are needed to address the inclusion of cN+ for risk-stratification model.

In the present study, type of urinary diversion was selected one of independent predictors for disease recurrence. This result suggested possibilities that the strong selection bias for urinary diversion and/or opportunity difference for surveillance after surgery. There is a strong selection bias for orthotopic bladder substitution including younger age, good performance status, and lower-risk for local recurrence. Due to the retrospective analysis, we could not assess the influence of frequent care in patients with stoma might have impact of follow-up surveillance. Further prospective studies are necessary to address these important clinical questions.

The use of neoadjuvant chemotherapy (NAC) for patients with renal impairment remains unclear [29–33]. Based on several guidelines [34, 35] NAC was not recommended in the patients with CKD (= cisplatin-unfit) and carboplatin-based regimen was believed less effective than cisplatin. However, no clear evidence currently supports the superiority of cisplatin-based regimens over carboplatin-based regimens in the neoadjuvant setting [36]. Therefore, we used short-term (two courses) carboplatin-based NAC for patients with CKD, and performed RC within 90 days [37]. Indeed, our previous studies with short-term carboplatin-based NAC with immediate cystectomy suggested potential benefit of carboplatin based NAC for MIBC patients with preoperative CKD [30, 32, 33, 38]. Based on these results, although two NAC courses might be insufficient, we believe our protocol did not harm to prognosis in MIBC patients with CKD.

Although use of this risk score-based protocol may reduce over evaluation, the cost was still substantial because of the low relapse rate in the low-risk group. This result highlights the limitation of predicting relapse risk exclusively on clinical information. For a better understanding of tumor biology, biomarkers that predict malignant potential are necessary. Genome-based molecular classification is a potential biomarker [39, 40]. A basal MIBC type has been associated with decreased disease-specific and overall survival because of its invasiveness and metastatic potential at presentation [39]. There is an urgent need to identify a molecular biomarker to inform clinical management.

This study was limited by its retrospective design. First, as we could not control all variables, it was subject to selection bias and the influence of confounding factors including all medical costs for oncological surveillance, CT cost for with or without a contrast medium, numbers of dissected and positive lymph nodes, smoking status, regimen of NAC and the influence of adjuvant/salvage therapy. In addition, we could not address the optimal timing and utility of urine cytology for routine screening in the present study because only five patients (2.9%) were detected by urine cytology. Furthermore, we could not control the influence of frequent care in patients with stoma that might have impact of follow-up surveillance. Second, we focused two points; risk stratification and cost effectiveness of postoperative follow-up. In this point of view, it might be hard to discuss these two issues at the same time. However, when we simply evaluated the medical cost based on guideline recommended pathological risk factors, we found that pathology-based protocol did not improve the cost-effectiveness. In addition, it is not optimal for a clinical practice to use all previously reported risk factors. Therefore, we selected risk factors that significantly associated with tumor progression in our patients, and then developed the optimal risk stratification and surveillance protocol. Third, we could not clearly explain why CVD and CKD were selected as significant factors for recurrence in MIBC patients who underwent RC. Because there are not enough evidences to support the clinical implication of CVD and CKD for tumor progression, further basic and clinical research are necessary. Forth, the risk score-based surveillance model might increase the number of patients not detected by screening. Finally, the implication of this study may not suitable for other population such as non-Asian people because the entire Japanese population is covered by universal health insurance (maximum copayment of 10% to 30%). However, it is not easy to make the one-fit-all surveillance protocol due to medical system differences among the nations. Therefore, our study provides the idea that risk stratification in each nation is necessary to improve the cost-effectiveness in each medical system.

Despite these limitations, this study adds to our knowledge of the cost effectiveness of oncological surveillance after RC. Although the importance of risk-stratified surveillance protocol has been reported, no study previously reported cost-effectiveness calculation using risk-score based protocol. We believe our study provides the idea for risk stratification to improve the cost-effectiveness. A prospective study on the cost effectiveness of follow-up using a universal, standard, and easily applicable surveillance model is required to validate these results.

In conclusion, a risk-score-stratified surveillance protocol has the potential to reduce over investigation during follow-up making surveillance more cost effective. Further study is needed to determine the impact of risk-stratification on the cost effectiveness of oncological follow-up after RC.

## MATERIALS AND METHODS

### Ethics statement

This retrospective study was performed following the ethical standards of the Declaration of Helsinki, and was approved by the Ethics Committee of the Hirosaki University School of Medicine (authorization numbers 2015–258 and 2016–225).

### Patient selection

Between May 1996 and December 2016, a consecutive series of 581 adults underwent RC and urinary diversion at Hirosaki university hospital, Aomori Rosai Hospital, Mutsu General Hospital, and Aomori Prefectural Central Hospital.

### Patient variables

The variables analyzed were age, sex, Eastern Cooperative Oncology Group performance status (ECOG PS), clinical stage, renal function, and history of hypertension (HTN), cardiovascular disease (CVD), and diabetes mellitus (DM). CVD was defined as a positive history of cardiac surgery, angina, myocardial infarction, stroke, or taking any cardiotonic agents and/or coronary vasodilators. Diabetic patients were defined as those with a history of type 2 diabetes or those who met the relevant diagnostic criteria and required glycemic control. Renal function was evaluated by estimated glomerular filtration rate (eGFR) using a modified version of the abbreviated Modification of Diet in Renal Disease Study formula for Japanese patients [41]. Chronic kidney disease (CKD) criteria included a preoperative eGFR < 60 mL/min/1.73 m^2^. Tumor stage and grade were defined by the 2009 TNM classification of the Union of International Cancer Control [42]. We used computed tomography (CT) and cystoscopy for preoperative TNM classification. Magnetic resonance imaging (MRI) and bone scintigraphy were indicated for the selected patients with when advanced disease (≥T3b and/or cN+) was suspected. Postoperative complications were evaluated by the Clavien–Dindo classification [43].

### Neoadjuvant chemotherapy (NAC)

We routinely used 2 or 3 NAC regimens for MIBC patients after 2005. The exclusion criteria for NAC in our institute include patients with chronic active hepatitis, severe liver dysfunction, severe chronic obstructive pulmonary disease with home oxygen therapy, severe chronic heart failure, or undergoing dialysis. These were a platinum-based combination regimens using either gemcitabine plus cisplatin (GCis), gemcitabine plus carboplatin (GCb), or methotrexate, vinblastine, adriamycin, and cisplatin (MVAC). Regimens were selected based on guidelines regarding eligibility for the proper use of cisplatin [44] and a patient’s overall status.

### Surgical procedure

All patients experienced RC, urinary diversion, and a standard pelvic lymph node dissection (PLND) that included the removal of the obturator, external iliac, hypogastric, and common iliac lymph node chains (there were no para-aortic or paracaval dissections). All RCs were performed by high-volume surgeons (> 15 cases per year, or >100 cases in recent 10 years) using the same basic technique [45]. Orthotopic ileal neobladder construction, ileal conduit diversion, and cutaneous ureterostomy were performed by previously reported methods [46–48].

### Follow-up surveillance protocol

Oncological follow-up after RC was performed following National Comprehensive Cancer Network and European Association of Urology guidelines and previously published pathology protocols [4, 9]. Based on pathological outcome after RC, all patients were stratified for oncological follow-up to normal-risk (≤ pT2N0) and high-risk (≥ pT3N0, pN+, lymphovascular invasion positive (LVI+), or histological variants) groups. The pathology-based protocol is shown in Table 1 (upper rows). Follow-up of high-risk patients was recommended every 3 months for the first 2 years after surgery, semiannually for the next 3 years, and annually thereafter, barring evidence of disease recurrence. Follow-up of normal-risk patients was recommended every 3 months for the first year and every 6 months for the following 3 years and annually thereafter in patients without any evidence of disease recurrence. Follow-up evaluation included urine cytology and ultrasonography every 3–6 months to monitor urothelial recurrence and hydronephrosis in both groups. Blood biochemistry, and CT of the chest/abdomen/pelvis were performed every 3–6 months for at least 5 years to monitor serum electrolytes, blood urea nitrogen, serum creatinine, and liver function. Evaluations by bone scans or brain imaging were performed when clinically indicated. Disease recurrence was classified as in the lymph nodes, visceral organs, local pelvis, bones, urothelium (urethra plus upper urinary tract), or brain. Lymph node recurrence included metastasis to local pelvic, paraaortic, thoracic, mediastinal, and paratracheal lymph nodes. Visceral organ recurrence included metastasis to the liver, lungs, adrenal glands, and other intra-abdominal organs. The first recurrence after RC was recorded.

### Adjuvant and salvage therapy after radical cystectomy

Adjuvant chemotherapy was not routinely administered. Systemic chemotherapy after recurrence consisted of a platinum-based combination regimen with GCis, GCb, gemcitabine, carboplatin and docetaxel (GCD), docetaxel, ifosfamide and nedaplatin (DIN), or MVAC. Regimens were selected based on residual renal function and overall status. Vital status was identified from death certificates or physician correspondence. For patients followed elsewhere, the cystectomy registry at our institution collected annual outcomes from the patient and treating physician.

### Outcome measurements

Recurrence-free survival, time to recurrence, and estimated cost per one recurrence detection by the pathology-based protocol were recorded for the normal- and high-risk groups. To estimate cost-benefit, we calculated the medical cost of follow-up to detect one recurrence (= [screening cost in a follow-up period] / [number of patients with recurrence]) using an exchange rate of 100 yen to the U.S. dollar. Estimated medical costs were $350 for a CT, $70 for blood testing, $53 for ultrasonography, and $25 for urine cytology. The cost of prescriptions, medications, and doctor fees were not included in the analysis. To compare cost effectiveness, we developed a novel risk score-based protocol using a multivariate Cox proportional hazard regression model. Patient risk-scores were calculated by summing the number of independent risks suggested in the multivariate analysis, and the patients were stratified into low (0-1), intermediate (2–3), and high (4–6) (Table 4). Then, we developed our ad-hoc surveillance protocol using real incidences of recurrence after radical cystectomy (Table 1, lower columns). When the incidence of recurrence was frequent at a certain period, we defined the routine screening is necessary in this period. On the other hand, when the incidence of recurrence was not frequent (0 or 1) at a certain period, we defined the routine screening is not necessary in this period. Based on this rule, we developed our surveillance protocol that enhancing the cost-effectiveness without increasing the number of patients who were not detected by screening. The estimated cost per one recurrence detection in the pathology-based and risk score-based protocols were compared.

### Statistical analysis

Statistical analysis was performed using SPSS version. 24.0 (SPSS, IBM Japan, Tokyo, JPN) and GraphPad Prism 5.03 (GraphPad Software, San Diego, CA, USA). Categorical variables were compared using Fisher’s exact test or the chi-square test. Differences between groups were compared using *t*-tests for normally distributed values or the Mann-Whitney *U*-test for values that were not normally distributed. The Kruskal-Wallis test was used to compare medians among three groups. All tests were two-sided, and a *P* value < 0.05 was considered statistically significant. Overall survival in patients with recurrence, stratified by risk criteria was estimated using the Kaplan–Meier method and compared by the log rank test. Cox proportional hazard regression models were used to identify factors independently associated with recurrence-free survival; hazard ratios (HRs) with 95% confidence intervals (CIs) were calculated after controlling simultaneously for potential confounders, including patient demographic and clinicopathological variables. Model variables included age, sex, ECOG PS, history of HTN, DM, CVD, preoperative CKD, NAC, urinary diversion, ≥ pT3 or SM+, pN+, LVI+, and nonurothelial carcinoma components.

### Ethical standards

This study was performed in accordance with the ethical standards of the Declaration of Helsinki and approved by an ethics review board of Hirosaki University School of Medicine (authorization numbers; 2016–225 and 2015–258).
